# Effect of the transcutaneous electrical stimulation system on esophageal-acid exposure in patients non-responsive to once-daily proton-pump inhibitor: proof-of-concept study

**DOI:** 10.1093/gastro/goab002

**Published:** 2021-02-11

**Authors:** Ram Dickman, Sigal Levy, Tsachi Tsadok Perets, Maor Hazani-Pauker, Doron Boltin, Hemda Schmilovitz-Weiss, Issa Nidal, Matan Siterman, Dan Carter, Ronnie Fass, Rachel Gingold-Belfer

**Affiliations:** 1 Department of Gastroenterology, Rabin Medical Center, Beilinson Campuses, Petah Tikva and Sackler Faculty of Medicine, Tel Aviv University, Tel Aviv, Israel; 2 Statistics Education Unit, Academic College of Tel Aviv-Yafo, Tel Aviv-Yafo, Israel; 3 Department of Surgery B, Rabin Medical Center, Hasharon Campus, Petah Tikva and Sackler Faculty of Medicine, Tel Aviv University, Tel Aviv, Israel; 4 Division of Gastroenterology and Hepatology, Chaim Sheba Medical Center, Ramat Gan and Sackler Faculty of Medicine, Tel Aviv University, Tel Aviv, Israel; 5 Division of Gastroenterology and Hepatology, Metrohealth Medical Center, Cleveland, OH, USA

**Keywords:** gastroesophageal reflux disease, transcutaneous electrical stimulation system, proton-pump inhibitor

## Abstract

**Background:**

Gastroesophageal reflux disease (GERD) is a common disorder. Overall, ≤35% of GERD patients fail the standard dose of proton-pump-inhibitor (PPI) treatment. Due to the high prevalence and low satisfaction rate with treatment failure, there is an unmet need for new treatment. Our aim was to evaluate whether the use of the transcutaneous electrical stimulation system (TESS) can reduce esophageal-acid exposure in GERD patients unresponsive to standard-dose PPI.

**Methods:**

We enrolled 10 patients suffering from heartburn and regurgitation with an abnormal esophageal-acid exposure (off PPIs) who failed standard-dose PPI. After the placement of a wireless esophageal pH capsule, all patients were treated with TESS. The primary end point was the reduction in the baseline (pretreatment) 24-hour percent total time pH <4 and/or DeMeester score by 50%.

**Results:**

Seven GERD patients (five females and two males, aged 49.3 ± 10.1 years) completed the study. At baseline, the mean percent total time pH <4 was 12.0 ± 4.9. Following TESS, the mean percent total time pH <4 dropped to 5.5 ± 3.4, 4.5 ± 2.6, 3.7 ± 2.9, and 4.4 ± 2.5 on Days 1, 2, 3, and 4, respectively. At baseline, the mean DeMeester score was 39.0 ± 18.5. After TESS, the mean DeMeester score dropped to 15.8 ± 9.2, 13.2 ± 6.8, 11.2 ± 9.4, and 12.0 ± 6.8 on Days 1, 2, 3, and 4, respectively.

**Conclusion:**

TESS is a safe and potentially effective modality in reducing esophageal-acid exposure in GERD patients unresponsive to standard-dose PPI. A larger and prospective controlled study is needed to verify these preliminary results.

## Introduction

Gastroesophageal reflux disease (GERD) is a chronic, persistent, and common medical problem with a pooled worldwide prevalence of ∼13% [1, 2]. Proton-pump-inhibitor (PPI) therapy is the most effective treatment for GERD due to its profound effect on acid secretion, resulting in symptom control, esophageal healing, and prevention of complications. However, despite its effectiveness, PPI failure is very common in GERD, affecting ≤44% of patients [3]. Several mechanisms have been proposed to account for symptom generation in patients who fail to respond to PPI treatment. First and foremost, it is necessary to exclude poor compliance and adherence [3–5], which are driven by many factors including concerns regarding PPI side effects, such as osteoporosis [6], dementia, vitamin and mineral deficiencies [7], and the risk of gastric cancer in Western populations [8]. Other underlying mechanisms that may account for the lack of response to PPIs include overlap with a functional esophageal disorder, residual reflux, gastroparesis, and functional bowel disorder.

In the case of failure on standard doses of PPI, current first-line recommendations are to ensure adherence and compliance with treatment, doubling the PPI daily dosage, switching to another brand of PPI, or adding a supplemental antireflux medication [5]. However, Fass *et al*. [9] found that the therapeutic gain of doubling the PPI dose was limited. In the case of PPI failure, there are currently no satisfactory non-invasive treatment options. Laparoscopic fundoplication is a possible therapeutic modality for patients with proven pathologic acid exposure despite doubling the dose of PPI. Nevertheless, <1% of eligible patients eventually undergo fundoplication due to its invasive nature and related side effects [10–14].

The transcutaneous electrical stimulation system (TESS) is a novel device developed by GerdCare Medical™ (Yokneam, Israel) for the treatment of GERD. To date, no studies have been performed to assess the effect of TESS on esophageal-acid exposure and symptoms in patients who continued to be symptomatic on standard-dose PPI. Therefore, the aim of this proof-of-concept study was to determine the efficacy of TESS on esophageal-acid exposure and symptoms in patients with GERD who were unresponsive to standard-dose PPI. Our hypothesis was that TESS would be effective, safe, and with minimal adverse effects.

## Patients and methods

### Study subjects

We included adult patients (age >18 years) with typical GERD-related symptoms (heartburn and/or regurgitation), >3 days per week, over a period of 3 months prior to screening. All patients reported heartburn or regurgitation while on omeprazole 20 mg once daily and demonstrated abnormal esophageal-acid exposure off PPI therapy. We defined the abnormal pretreatment esophageal-acid exposure off PPI as baseline pH.

All eligible patients were referred by gastroenterologists from outpatient clinics of two major safety-net hospitals. Exclusion criteria included gastric or esophageal surgery, active peptic-ulcer disease, malignancy, pregnancy, uncontrolled diabetes mellitus, severe cardiac disorders, implanted electrical devices including cardiac pacemaker or defibrillator, skin allergy to patches, severe pulmonary disease, body mass index >30 kg/m^2^, gastroparesis, treatment with narcotics, reluctance or incapability to provide informed consent, inability to fully complete all phases of the study, and contraindication for wireless pH capsule placement, i.e. need to undergo MRI 2 weeks after the procedure. Patients with esophageal varices, erosive esophagitis, Barrett's esophagus, and peptic stricture were also excluded from the study. To ensure that all patients had an abnormal esophageal-acid exposure at baseline, eligible patients had to have undergone ambulatory 24-hour pH study off PPI within the last year, with a percent total time pH <4 and a DeMeester score >4.2 and >14.7, respectively.

### Study design

This prospective, proof-of-concept trial was conducted between January 2016 and May 2017, in accordance with the principles of the Declaration of Helsinki, Good Clinical Practice and was approved by the Human Subjects Protection Program of the Rabin Medical Center, Petah Tikva, Israel (clinical.trial.gov registry NCT02500264). All patients provided written informed consent before enrollment into the study.

Our study design included two phases. The first phase consisted of a 3-week trial planned to identify the optimal pulse parameters (current and frequency) and the position of the TESS electrodes (out of four different protocols), based on the effects of TESS on symptoms (heartburn and regurgitation) and esophageal-acid exposure. The second phase consisted of a 4-week trial, planned to evaluate the efficacy of TESS by assessing the **s**ymptom-severity score and quality of life.

**Figure 1.**
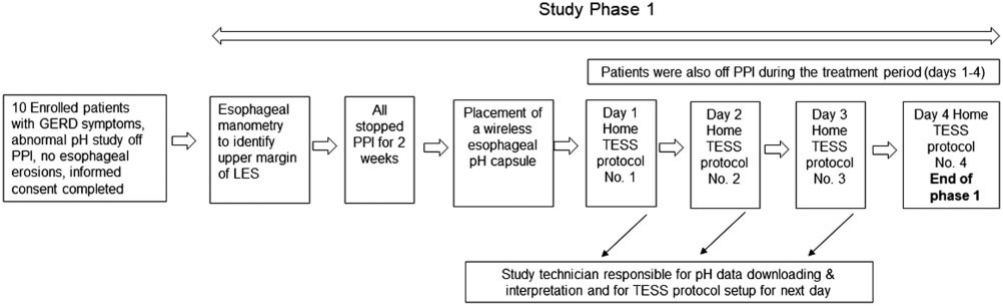
Phase 1 study design. GERD, gastroesophageal reflux disease; PPI, proton-pump inhibitor; LES, lower esophageal sphincter; TESS, transcutaneous electrical stimulation system.

The first phase (3-week trial) was divided into two sub-periods: during the first 2-week baseline period, patients with classic GERD-related symptoms, non-erosive reflux disease (NERD), who did not respond to treatment with PPI once a day underwent esophageal manometry to identify the proximal border of the lower esophageal sphincter (LES). Patients discontinued PPIs during this period. During the subsequent 1-week treatment period, patients underwent wireless pH capsule (Bravo™, Medtronic, Minneapolis, USA) placement and monitoring. Patients were then assigned to four consecutive home-based TESS protocols, supervised consecutively every 24 hours by a dedicated study medical technician (A.M.). Phase 1 of the study design, with all temporal steps, is shown in Figure 1. Each protocol comprised specific pulse parameters (current and frequency) and different positioning of the TESS electrodes.

**Figure 2.**
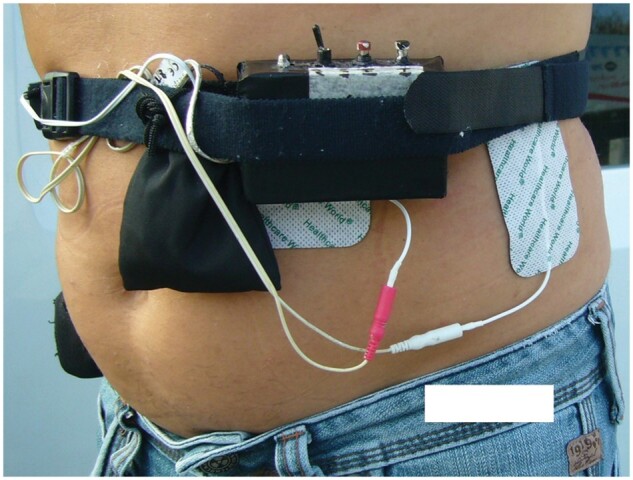
The transcutaneous electric stimulation device (TESS) is applied by a technician to the patient's abdominal wall and electrically stimulates the abdominal muscles

At each visit, the medical technician was responsible for downloading and analysing pH data from the previous day and repositioning the device for the next 24 hours (Figure 2).

### Study equipment and measurements

High-resolution esophageal manometry (HRM) is a diagnostic clinical tool to evaluate esophageal function. HRM uses a 36-channel, high-resolution catheter to transmit intraluminal pressure data that are subsequently converted into dynamic esophageal pressure topography plots.

The wireless pH capsule system (Bravo™, Medtronic, Minneapolis, USA) records esophageal pH data for ≤96 hours [15]. Patients were instructed to complete a personal diary recording their meal times, position changes, symptom times, and characteristics. Data analysis was performed every 24 hours by a technician using commercially available computer software (Bravo™, Medtronic, Minneapolis, USA).

**Figure 3.**
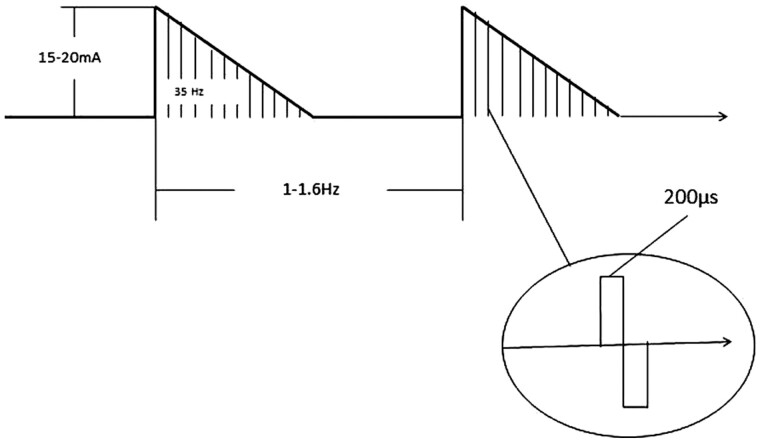
The transcutaneous electric stimulation device (TESS) stimulates the abdominal muscles with modulated asymmetrical biphasic current waveforms (sawtooth wave) with a typical controlled current level of 15–40 mA base to pick and maximum 70 mA. A high-voltage generator generates 120 V to the current contorted circuit (not shown in the figure). Each waveform includes a burst of multiple rectangular biphasic pulses with a duration of 200 µs and frequency of 35 Hz. During the study, two modulated burst frequencies of 1 and 1.6 Hz were tested.

The TESS (GerdCare Medical™, Yokneam, Israel) is a specifically designed, non-invasive device prototype (concept model) for GERD treatment. TESS comprises a sealed plastic case that contains a pulse generator and rechargeable batteries; a pair of replaceable electrodes coated with a conductive adhesive hydrogel are connected via conductive wires to the case. This device also contains a belt with a piezoelectric breathing sensor for synchronizing the stimulation pulses with the breathing phase (Figure 2). Thus, the generated pulses are synchronized with the patient’s breathing phase (active during inhalation). The TESS stimulates the abdominal muscles with modulated asymmetrical biphasic current waveforms (sawtooth wave) with a typical controlled current level of 15–40 mA base to pick and maximum 70 mA. A high-voltage generator generates 120 V to the current contorted circuit (not shown in the figure). Each waveform includes a burst of multiple rectangular biphasic pulses with a duration of 200 µS and frequency of 35 Hz. During the study, two modulated burst frequencies of 1 and 1.6 Hz were tested (Figure 3). The device was applied to the abdominal wall in four rotating positions. The user has the possibility to control the stimulation parameters via internal keys or via a special application.

TESS has immediate effects and may have a long-term effect:

Immediate effects: The asymmetric electric stimulation bursts generate asymmetrical contractions of the muscles. This mechanism was previously reported by several studies that investigated mucous transport within the bronchial tree [16–19]. Such dynamic asymmetrical movements are equivalent to the well-known mechanism of the *Vibrating Conveyer* that is used in various industrial applications [20–22]. As the static friction is higher than the dynamic friction, materials (solids or liquids) move toward the direction of the slow slope. In this study, we applied the same principle in the hope of generating movements of the refluxate from the esophagus to the stomach, thereby reducing esophageal-acid exposure.

Possible long-term effect: LES modulation. The electrical pulses generate abdominal-muscle contractions that ultimately may increase the LES resting pressure and decrease transient LES relaxation, thus reducing esophageal-acid exposure.

### Efficacy assessment

The primary end point was significant, with a >50% reduction in the symptom-severity score and esophageal-acid exposure compared to the baseline (pretreatment). We selected a validated questionnaire for the assessment of symptoms at baseline compared to those in the treatment period. For the second outcome, we used two validated parameters: percent total time pH <4 and the DeMeester score. These two parameters were assessed every 24 hours for ≤4 consecutive days in all seven patients included in the final analysis. The secondary end point was to determine patients’ compliance with study phases.

### Tolerability and safety assessments

Adverse events were assessed by the technician who repositioned the TESS every 24 hours during the treatment phase.

### Statistical analysis

Due to the small sample size, we used a nonparametric approach in our data analysis. Data analysis was performed using the SPSS version 25.0. Statistics were calculated using the Wilcoxon signed-rank test to evaluate the reduction in esophageal-acid exposure. We considered *P *<* *0.05 as statistically significant, yet we noted marginally significant results (*P *<* *0.1) as well for better understanding of the results.

## Results

A total of 10 patients were eligible for enrollment into this study and 7 (70%) completed the first phase of the study. At the beginning of the first phase, we did not identify any significant differences between the four predesigned protocols of TESS (pulse current and frequency) and the position of the electrodes. Consequently, we stopped manipulating the stimulation modes and position of the electrodes. Symptom-severity scores were not assessed during the first phase of this study due to the short treatment period (4 days). Three patients were excluded from the study: two because of technical issues related to the use of the pH recorder (removed or disconnected) and one due to incorrect use of the device’s belt. Therefore, a total of seven patients were included in the final analysis of esophageal-acid exposure before and after treatment with TESS.

All patients had a normal upper endoscopy while on standard-dose PPI. The mean age of the subjects was 49.1 ± 10.1 years and 71% of the patients were female. The mean duration of symptoms was 4 years. Demographic characteristics and esophageal-acid exposure, assessed by percent total time pH <4 at baseline and on Days 1, 2, 3, and 4, are described in **Table 1**.

**Table 1.** Demographic characteristics and esophageal-acid exposure, assessed by percent total time pH <4 at baseline and on Days 1, 2, 3, and 4

Percent total time pH <4					Gender	Age, years	Patient
Day 4	Day 3	Day 2	Day 1	Baseline pH			
4%	4%	3%	10%	18%	Male	45	1
1%	2%	2%	2%	5%	Female	30	2
4%	6%	9%	11%	11%	Female	67	3
^*^	1%	3%	4%	11%	Female	39	4
5%	9%	6%	9%	9%	Female	47	5
22%	28%	29%	^*^	64%	Female	55	6
^*^	1%	4%	6%	3%	Male	61	7

* Missing data.

Baseline pH refers to the abnormal pretreatment (or known) esophageal-acid exposure.

### Primary endpoint

**Figure 4.**
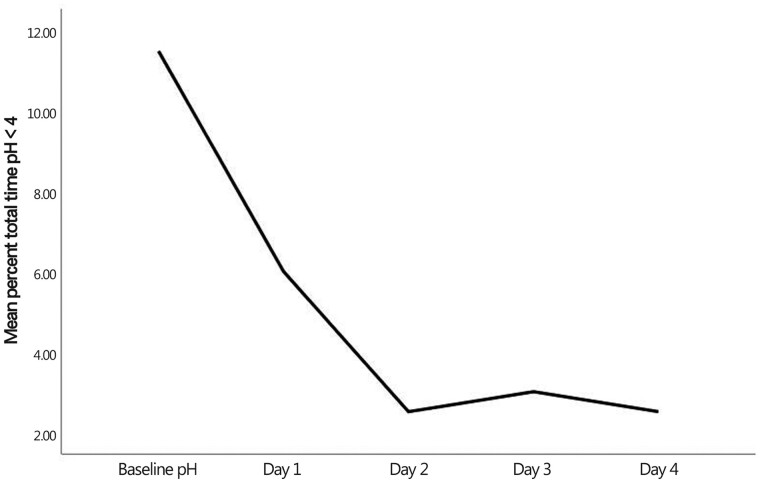
The decline curve of the mean percent total time pH <4 from baseline to Day 4

**Figure 5.**
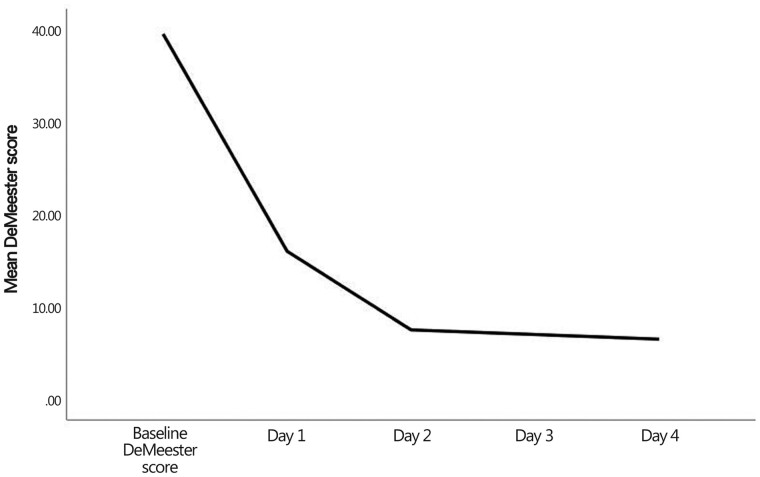
The decline curve of the mean DeMeester score from baseline to Day 4

Wilcoxon test showed a significant decrease in the percent total time pH <4 between baseline and Day-3 and -4 measurements (*Z *=* *2.2, *P *=* *0.027 and *Z *=* *2.0, *P *=* *0.043, respectively). Figure 4 demonstrates the decline curve in the mean percent total time pH <4 from baseline to Day 4. Moreover, the Wilcoxon test revealed a significant decrease in DeMeester scores from baseline to Days 3 and 4 (*Z *=* *2.2, *P *=* *0.028 and Z = 2.0, *P *=* *0.043, respectively). Figure 5 demonstrates the decline curve of the mean DeMeester score from baseline to Day 4.

### Secondary endpoint

In all seven patients who completed the study, compliance and adherence were 100%.

### Tolerability and safety assessments

No adverse events were reported during the first phase of this study. TESS did not disturb sleep and was well tolerated by all patients.

## Discussion

PPI failure in GERD patients presents a major diagnostic and therapeutic challenge [23]. The combination of a very prevalent chronic problem and a high level of treatment failure has prompted a search for novel therapeutic approaches. Identifying validated and safe alternative therapies for GERD is clearly needed in patients who continue to experience GERD symptoms despite high doses of PPI and for those who decline medical or surgical treatment [13, 24].

Our intention was to assess a non-invasive device that stimulates abdominal muscles and thereby triggers esophageal clearance without affecting gastric-acid production. This is the first study to explore the effect of TESS on esophageal-acid exposure among patients who have failed standard-dose PPI. Our results demonstrated that TESS is a safe, well tolerated, and effective device. All seven (100%) patients who completed the study reached the primary end point of 50% reduction in esophageal-acid exposure.

Compared to the electrical-stimulation-therapy (EST) studies that investigated laparoscopically implanted electrodes in the LES area [25, 26], we used a non-invasive stimulation system that does not require hospitalization. TESS uses a piezoelectric sensor in which the stimulation pulse is delivered in synchronization with the breathing phase throughout the day and not intermittently like EST [25]. Finally, we found that TESS has the ability to reduce esophageal-acid exposure in a very short period of time. TESS is assumed to induce vibrations, generating clearance of the refluxate from the esophagus to the stomach, hence reducing esophageal-acid exposure.

The strengths of this study were the highly selected patient population, and the simple and clear outcome measure. In addition, by using a wireless device to measure acid exposure at home, we could minimize changes in lifestyle or eating behaviors.

Limitations of this study were the small sample size; unequal sex distribution, as most of our patients were females; and the lack of a controlled group (e.g. a sham-treatment arm) or a day-on–day-off design. In our ongoing second phase of this study, we compare acid exposure on Day 1 when TESS is not functioning with the rest of the 3 days when TESS is functioning. The small number of participants was due to our difficulty in identifying and enrolling symptomatic patients with a true NERD (typical symptoms, no esophageal erosions, and an abnormal esophageal-acid exposure) who were non-responsive to once-daily PPI treatment. It is now recognized that the cumulative incidence of functional heartburn is quite high among patients with NERD. Consequently, the number of patients who were diagnosed with true NERD, fulfilled inclusion and exclusion criteria, and consented to enroll into this study was lower than expected. In order to increase patient recruitment, in our second-phase ongoing study, we include true NERD who are unresponsive and responsive to once-daily PPI as well as to double-dose PPI treatment. Furthermore, outcome variables, such as improvement in heartburn, regurgitation, and health-related quality of life, were not assessed due to the short treatment period. Those clinical parameters should be evaluated in future studies including our ongoing second phase of this study and in randomized–controlled clinical trials. Another limitation of this study regards esophageal-acid exposure in the supine and upright positions as well as during the post-prandial periods. Unfortunately, not all the patients completed their diaries and therefore this information was not included in the analysis. Finally, esophageal peristalsis and the LES high-pressure zone were not assessed during TESS stimulation or at the end of the study. In our second-phase ongoing study, we will compare esophageal mechanical parameters at baseline with those at the end of the study. Despite the small size, our study suggested that TESS may be effective in reducing esophageal-acid exposure in patients who remained symptomatic despite standard-dose PPI treatment.

Our results provide justification for performing a larger controlled clinical trial to explore the mechanism of action of TESS and to study the effects of TESS on symptoms in patients with GERD and failure to respond to PPI [27–29].

In summary, TESS appears to be a safe and effective technique in reducing esophageal-acid exposure in GERD patients unresponsive to standard-dose PPI. A larger prospective, sham-controlled study is needed to verify these early results.

## Authors’ contributions

R.D., M.D.R., M.S., D.C., R.F., and R.G.-B. planned the study. R.D., M.D.R., M.S., D.C., and R.G-B. conducted the study. T.T.P., H.S.-W., and I.N. collected the data. S.L., T.T.P., H.S.-W., and I.N. interpreted the data. R.D. and R.F. drafted the manuscript. All authors read and approved the final manuscript.

## Funding

**None.**

## Conflicts of interest

None declared.

## References

[1] Eusebi LH, Ratnakumaran R, Yuan Y et al Global prevalence of, and risk factors for, gastrooesophageal reflux symptoms: a meta-analysis. Gut 2018;67:430–40. 28232473 10.1136/gutjnl-2016-313589

[2] Fass R, Fennerty MB, Vakil N. Nonerosive reflux disease: current concepts and dilemmas. Am J Gastroenterol 2001;96:303–14. 11232668 10.1111/j.1572-0241.2001.03511.x

[3] Dickman R, Boaz M, Aizic S et al Comparison of clinical characteristics of patients with gastroesophageal reflux disease who failed proton pump inhibitor therapy versus those who fully responded. J Neurogastroenterol Motil 2011;17:387–94. 22148108 10.5056/jnm.2011.17.4.387 PMC3228979

[4] Tack J, Koek G, Demedts I et al Gastroesophageal reflux disease poorly responsive to single-dose proton pump inhibitors in patients without Barrett’s esophagus: acid reflux, bile reflux, or both? Am J Gastroenterology 2004;99:981–8. 10.1111/j.1572-0241.2004.04171.x 15180713

[5] Richardson P, Hawkey CJ, Stack WA. Proton pump inhibitors: pharmacology and rationale for use in gastrointestinal disorders. Drugs 1998;56:307–35. 9777309 10.2165/00003495-199856030-00002

[6] Ngamruengphong S, Leontiadis GI, Radhi S et al Proton pump inhibitors and risk of fracture: a systematic review and meta-analysis of observational studies. Am J Gastroenterol 2011;106:1209–18. 21483462 10.1038/ajg.2011.113

[7] Kwok CS, Arthur AK, Anibueze CI et al Risk of clostridium difficile infection with acid suppressing drugs and antibiotics: meta-analysis. Am J Gastroenterol 2012;107:1011–9. 22525304 10.1038/ajg.2012.108

[8] Wan QY, Wu XT, Li N et al Long-term proton pump inhibitors use and risk of gastric cancer: a meta-analysis of 926 386 participants. Gut 2019;68:762–4. 29615489 10.1136/gutjnl-2018-316416

[9] Fass R, Murthy U, Hayden CW et al Omeprazole 40 mg once a day is equally effective as lansoprazole 30 mg twice a day in symptom control of patients with gastro-oesophageal reflux disease (GERD) who are resistant to conventional-dose lansoprazole therapy: a prospective, randomized multi-center study. Aliment Pharmacol Ther 2000;14:1595–603. 11121907 10.1046/j.1365-2036.2000.00882.x

[10] Reynolds JL, Zehetner J, Nieh A et al Charges, outcomes, and complications: a comparison of magnetic sphincter augmentation versus laparoscopic Nissen fundoplication for the treatment of GERD. Surg Endosc 2016;30:3225–30. 26541730 10.1007/s00464-015-4635-6

[11] Richter JE. Gastroesophageal reflux disease treatment: side effects and complications of fundoplication. Clin Gastroenterol Hepatol 2013;11:465–7. 23267868 10.1016/j.cgh.2012.12.006

[12] Subramanian CR, Triadafilopoulos G. Refractory gastroesophageal reflux disease. Gastroenterol Rep 2015;3:41–53. 10.1093/gastro/gou061 PMC4324866 25274499

[13] Gyawali CP, Fass R. Management of gastroesophageal reflux disease. Gastroenterology 2018;154:302–18. 28827081 10.1053/j.gastro.2017.07.049

[14] Pandolfino J, Lipham J, Chawla A et al A budget impact analysis of a magnetic sphincter augmentation device for the treatment of medication-refractory mechanical gastroesophageal reflux disease: a United States payer perspective. Surg Endosc 2020;34:1561–72. 31559575 10.1007/s00464-019-06916-6

[15] Wong WM, Bautista J, Dekel R et al Feasibility and tolerability of transnasal/per-oral placement of the wireless pH capsule vs. traditional 24-h oesophageal pH monitoring: a randomized trial. Aliment Pharmacol Ther 2005;21:155–63. 15679765 10.1111/j.1365-2036.2005.02313.x

[16] Chang HK, Weber ME, King M. Mucus transport by high-frequency nonsymmetrical oscillatory airflow. J Appl Physiol (1985) 1988;65:1203–9. 3182490 10.1152/jappl.1988.65.3.1203

[17] Lechtzin N, Wolfe LF, Frick KD. The impact of high-frequency chest wall oscillation on healthcare use in patients with neuromuscular diseases. Annals ATS 2016;13:904–9. 10.1513/AnnalsATS.201509-597OC 26999271

[18] Tatkov S, Pack RJ. Symmetrical-waveform high-frequency oscillation increases artificial mucus flow without changing basal mucus transport in in vitro ovine trachea. Respir Care 2011;56:435–41. 21255499 10.4187/respcare.00809

[19] Freitag L, Long WM, Kim CS et al Removal of excessive bronchial secretions by asymmetric high-frequency oscillations. J Appl Physiol (1985) 1989;67:614–9. 2793662 10.1152/jappl.1989.67.2.614

[20] Okabe S, Yokoyama Y, Boothroyd G. Analysis of vibratory feeding where the track has directional friction characteristics. Int J Adv Manuf Technol 1988;3:73–85.

[21] Winkler G. Analysing the hopping conveyor. Int J Mech Sci 1979;21:651–8.

[22] Sloot E, Kruyt N. Theoretical and experimental study of the transport of granular materials by inclined vibratory conveyors. Powder Technology 1996;87:203–10.

[23] Carlsson R, Galmiche JP, Dent J et al Prognostic factors influencing relapse of oesophagitis during maintenance therapy with antisecretory drugs: a meta-analysis of long-term omeprazole trials. Aliment Pharmacol Ther 1997;11:473–82. 9218069 10.1046/j.1365-2036.1997.00167.x

[24] Novotny M, Klimova B, Valis M. PPI long term use: risk of neurological adverse events? Front Neurol 2018;9:1142. 30671013 10.3389/fneur.2018.01142 PMC6331532

[25] Soffer E, Rodríguez L, Rodriguez P et al Effect of electrical stimulation of the lower esophageal sphincter in gastroesophageal reflux disease patients refractory to proton pump inhibitors. Wjgpt 2016;7:145–55. 26855821 10.4292/wjgpt.v7.i1.145 PMC4734948

[26] Stanak M, Erdos J, Hawlik K et al Novel surgical treatments for gastroesophageal reflux disease: systematic review of magnetic sphincter augmentation and electric stimulation therapy. Gastroenterol Res 2018;11:161–73. 10.14740/gr1024w PMC5997468 29915626

[27] Rodríguez L, Rodriguez P, Gómez B et al Two-year results of intermittent electrical stimulation of the lower esophageal sphincter treatment of gastroesophageal reflux disease. Surgery 2015;157:556–67. 25726315 10.1016/j.surg.2014.10.012

[28] DeMeester TR. Regarding “Two-year results of intermittent electrical stimulation of the lower esophageal sphincter treatment of gastroesophageal reflux disease.” Surgery 2015;158:1448. 25912380 10.1016/j.surg.2015.03.011

[29] Attwood S. Electrical stimulation for GERD: the need for controlled clinical trials. Surgery 2015;158:1449. 26189070 10.1016/j.surg.2015.05.007

